# Supplementary material for: Current Clinical Practice on the Management of Invasive Streptococcus Pyogenes Infections in Children: A Survey-Based Study
Source: Antibiotics (Basel). 2025 Sep 26;14(10):970. doi: 10.3390/antibiotics14100970 (PMC12561913; doi:10.3390/antibiotics14100970)
Supplement: Supplementary file 1 [file antibiotics-14-00970-s001.zip › antibiotics-3845928-supplementary.pdf]

Table S1.

SEPSIS				
QUESTION 1		LIKERT SCALE		
		SD/D	N A/D	A/SA
How much do you agree on a Likert scale with the indication to use each of the following options as first-line therapy for the treatment of bacteremia caused by GAS?	Penicillin	20.8%	0	79.1%
	Ampicillin	12.5%	4.2%	83.3%
	Ceftriaxon	20.8%	25%	54.1%
	Vancomycin	75%	16.7%	8.3%
QUESTION 2		LIKERT SCALE		
		SD/D	N A/D	A/SA
How much do you agree on a Likert scale with using combined antibiotic therapy (beta-lactam + other agent with activity on GAS) as first-line therapy to treat bacteremia caused by GAS?		41.7%	16.7%	41.6%
QUESTION 3		LIKERT SCALE		
		SD/D	N A/D	A/SA
If you disagree with the previous question, how much do you agree on a Likert scale with using combined antibiotic therapy (beta-lactam + other agent with activity on GAS) only in patients not responding to beta-lactams after 48-72 hours of treatment to manage bacteremia caused by GAS?		41.7%	8.3%	50%
QUESTION 4		LIKERT SCALE		
		SD/D	N A/D	A/SA
How much do you agree on a Likert scale with the indication to use each of the following options as combination therapy with beta-lactams for the treatment of bacteremia caused by GAS?	Clindamycin	0%	4.1%	95.8%
	Linezolid	29.1%	25%	45.8%
	Tedizolid	58.3%	25%	16.6%
	Vancomycin	58.2%	16.6%	24.9%
QUESTION 5				
Which of the following options do you prefer to define the best time to stop combination therapy (shifting to beta-lactam monotherapy) for the treatment of bacteremia caused by GAS?	48-72 h	8.3%		
	5-7 days	29.2%		
	I would continue combination therapy until the end of the treatment	8.3%		
	Until the patient is clinically and hemodynamically stable	54.2%		
QUESTION 6				

Which of the following options do you prefer to define the best time to complete antibiotic treatment for the treatment of bacteremia caused by GAS?	7-10 days, longer if a complicating deep-seated infection is detected	45.8%
	10-14 days, longer if a complicating deep-seated infection is detected	37.5%
	At least 14 days, longer if a complicating deep-seated infection is detected	16.6%
	Other	0%

STSS				
QUESTION 1		LIKERT SCALE		
		SD/D	N A/D	A/SA
How much do you agree on a Likert scale with the indication to use each of the following options as first-line therapy for the treatment of STSS caused by GAS?	Penicillin	20.8%	12.5%	66.6%
	Ampicillin	12.5%	12.5%	75%
	Ceftriaxon	16.6%	12.5%	70.7%
	Vancomycin	54.1%	8.3%	37.5%
QUESTION 2		LIKERT SCALE		
		SD/D	N A/D	A/SA
How much do you agree on a Likert scale with using combined antibiotic therapy (beta-lactam + other agent with activity on GAS) as first-line therapy to treat STSS caused by GAS?		20.8%	0%	79.2%
QUESTION 3		LIKERT SCALE		
		SD/D	N A/D	A/SA
If you disagree with the previous question, how much do you agree on a Likert scale with using combined antibiotic therapy (beta-lactam + other agent with activity on GAS) only in patients not responding to beta-lactams after 48-72 hours of treatment to manage STSS caused by GAS?		12.5%	62.4%	25%
QUESTION 4		LIKERT SCALE		
		SD/D	N A/D	A/SA
How much do you agree on a Likert scale with the indication to use each of the following options as combination therapy with beta-lactams for the treatment of STSS caused by GAS?	Clindamycin	0%	4.1%	95.8%
	Linezolid	20.8%	12.5%	66.6%
	Tedizolid	50%	20.8%	29.1%
	Vancomycin	50%	4.1%	45.8%
QUESTION 5				
Which of the following options do you prefer to define the best time to stop combination therapy (shifting to beta-lactam monotherapy) for the treatment of STSS caused by GAS?	48-72 h	12.5%		
	5-7 days	25%		
	I would continue combination therapy until the end of the treatment	20.8%		
	Until the patient is clinically and hemodynamically stable	41.6%		
QUESTION 6				

Which of the following options do you prefer to define the best time to complete antibiotic treatment for the treatment of STSS caused by GAS?	7-10 days, longer if a complicating deep-seated infection is detected	0%
	10-14 days, longer if a complicating deep-seated infection is detected	0%
	At least 14 days, longer if a complicating deep-seated infection is detected	83.3%
	Other	16.6%

NECROTIZING FASCITIS (NF)				
QUESTION 1		LIKERT SCALE		
		SD/D	N A/D	A/SA
How much do you agree on a Likert scale with the indication to use each of the following options as first-line therapy for the treatment of NF caused by GAS?	Penicillin	16.6%	8.3%	75%
	Ampicillin	16.6%	8.3%	75%
	Ceftriaxon	12.4%	20.8%	66.6%
	Vancomycin	41.6%	12.5%	45.8%
QUESTION 2		LIKERT SCALE		
		SD/D	N A/D	A/SA
How much do you agree on a Likert scale with using combined antibiotic therapy (beta-lactam + other agent with activity on GAS) as first-line therapy to treat NF caused by GAS?		0%	0%	100%
QUESTION 3		LIKERT SCALE		
		SD/D	N A/D	A/SA
If you disagree with the previous question, how much do you agree on a Likert scale with using combined antibiotic therapy (beta-lactam + other agent with activity on GAS) only in patients not responding to beta-lactams after 48-72 hours of treatment to manage NF caused by GAS?		0%	0%	0%
QUESTION 4		LIKERT SCALE		
		SD/D	N A/D	A/SA
How much do you agree on a Likert scale with the indication to use each of the following options as combination therapy with beta-lactams for the treatment of NF caused by GAS?	Clindamycin	0%	0%	99.9%
	Linezolid	16.6%	12.5%	70.8%
	Tedizolid	41.6%	33.3%	25%
	Vancomycin	45.8%	8.3%	45.8%
QUESTION 5				
Which of the following options do you prefer to define the best time to stop combination therapy (shifting to beta-lactam monotherapy) for the treatment of NF caused by GAS?	48-72 h	0%		
	5-7 days	20.8%		
	I would continue combination therapy until the end of the treatment	50%		
	Until the patient is clinically and hemodynamically stable	29.1%		
QUESTION 6				
Which of the following	10-14 days	8.3%		

options do you prefer to define the best time to complete antibiotic therapy for the treatment of NF caused by GAS in a pediatric patient?	Length of therapy depends on the clinical course and the adequacy of surgical debridement; therapy is usually continued for 14 days from the last positive culture obtained during surgical debridement	91.7%

PNEUMONIA				
QUESTION 1		LIKERT SCALE		
		SD/D	N A/D	A/SA
How much do you agree on a Likert scale with the indication to use each of the following options as first-line therapy for the treatment of GAS pneumonia?	Penicillin	20.8%	0%	79.1%
	Ampicillin	12.5%	4.1%	83.2%
	Ceftriaxon	12.5%	16.6%	70.8%
	Vancomycin	74.9%	12.5%	12.5%
QUESTION 2		LIKERT SCALE		
		SD/D	N A/D	A/SA
How much do you agree on a Likert scale with using combined antibiotic therapy (beta-lactam + other agent with activity on GAS) as first-line therapy to treat pneumonia caused by GAS?		54.2%	20.8%	24.9%
QUESTION 3		LIKERT SCALE		
		SD/D	N A/D	A/SA
If you disagree with the previous question, how much do you agree on a Likert scale with using combined antibiotic therapy (beta-lactam + other agent with activity on GAS) only in patients with GAS pneumonia and the following conditions?	Not responding after 48-72 h	4.2%	4.2%	91.6%
	Necrosis at chest XR/CT scan	0%	0%	100%
	Pleural effusion >2/3	12.5%	4.2%	83.2%
QUESTION 4		LIKERT SCALE		
		SD/D	N A/D	A/SA
How much do you agree on a Likert scale with the indication to use each of the following options as combination therapy with beta-lactams for the treatment of pneumonia caused by GAS?	Clindamycin	8.3%	8.3%	83.2%
	Linezolid	12.4%	8.3%	79.1%
	Tedizolid	49.9%	20.8%	29.1%
	Vancomycin	62.4%	4.1%	33.3%
QUESTION 5				
Which of the following options do you prefer to define the best time to stop combination therapy (shifting to beta-lactam monotherapy) for the treatment of pneumonia caused by GAS?	48-72 h	8.9%		
	5-7 days	37.5%		
	I would continue combination therapy until the end of the treatment	8.3%		
	When the patient is clinically and hemodynamically stable	45.8%		

<table>
<tr><th colspan="5">OTOMASTOIDITIS</th></tr>
<tr><td colspan="2" rowspan="2">QUESTION 1</td><td colspan="3">LIKERT SCALE</td></tr>
<tr><td>SD/D</td><td>N A/D</td><td>A/SA</td></tr>
<tr><td rowspan="4">How much do you agree on a Likert scale with the indication to use each of the following options as first-line therapy for the treatment of otomastoiditis caused by GAS?</td><td>Penicillin</td><td>25%</td><td>16.6%</td><td>58.2%</td></tr>
<tr><td>Ampicillin</td><td>16.6%</td><td>12.5%</td><td>70.7%</td></tr>
<tr><td>Ceftriaxon</td><td>8.3%</td><td>4.2%</td><td>87.4%</td></tr>
<tr><td>Vancomycin</td><td>58.2%</td><td>16.6%</td><td>25%</td></tr>
<tr><td colspan="2" rowspan="2">QUESTION 2</td><td colspan="3">LIKERT SCALE</td></tr>
<tr><td>SD/D</td><td>N A/D</td><td>A/SA</td></tr>
<tr><td rowspan="2">How much do you agree on a Likert scale with using combined antibiotic therapy (beta-lactam + other agent with activity on GAS) to treat otomastoiditis caused by GAS in following conditions?</td><td>As first line therapy</td><td>62.5%</td><td>20.8%</td><td>16.6%</td></tr>
<tr><td>Not responding after 48-72 h</td><td>12.5%</td><td>8.3%</td><td>79.2%</td></tr>
<tr><td colspan="2" rowspan="2">QUESTION 3</td><td colspan="3">LIKERT SCALE</td></tr>
<tr><td>SD/D</td><td>N A/D</td><td>A/SA</td></tr>
<tr><td rowspan="4">How much do you agree on a Likert scale with the indication to use each of the following options as combination therapy with beta-lactams for the treatment of otomastoiditis caused by GAS?</td><td>Clindamycin</td><td>8.3%</td><td>8.3%</td><td>83.2%</td></tr>
<tr><td>Linezolid</td><td>12.5%</td><td>16.6%</td><td>70.7%</td></tr>
<tr><td>Tedizolid</td><td>50%</td><td>16.6%</td><td>33.3%</td></tr>
<tr><td>Vancomycin</td><td>41.7%</td><td>4.2%</td><td>54%</td></tr>
<tr><td colspan="2">QUESTION 4</td><td colspan="3"></td></tr>
<tr><td rowspan="4">Which of the following options do you prefer to define the best time to stop combination therapy (shifting to beta-lactam monotherapy) for the treatment of otomastoiditis caused by GAS?</td><td>48-72 h</td><td colspan="3">8.3%</td></tr>
<tr><td>5-7 days</td><td colspan="3">37.4%</td></tr>
<tr><td>I would continue combination therapy until the end of the treatment</td><td colspan="3">33.3%</td></tr>
<tr><td>When the patient is clinically and hemodynamically stable</td><td colspan="3">20.8%</td></tr>
</table>

MENINGITIS				
QUESTION 1		LIKERT SCALE		
		SD/D	N A/D	A/SA
How much do you agree on a Likert scale with the indication to use each of the following options as first-line therapy for the treatment of meningitis caused by GAS?	Ampicillin	29.2%	16.6%	54%
	Ceftriaxon	8.3%	4.2%	95.8%
	Vancomycin	45.8%	20.8%	33.3%
QUESTION 2		LIKERT SCALE		
		SD/D	N A/D	A/SA
How much do you agree on a Likert scale with using combined antibiotic therapy (beta-lactam + other agent with activity on GAS) to treat meningitis caused by GAS in following conditions?	As first line therapy	29.2%	20.8%	50%
	Not responding after 48-72 h	33.3%	16.7%	50%
QUESTION 3		LIKERT SCALE		
		SD/D	N A/D	A/SA
How much do you agree on a Likert scale with the indication to use each of the following options as combination therapy with beta-lactams for the treatment of meningitis caused by GAS?	Clindamycin	45.8%	25%	29.2%
	Linezolid	16.6%	12.5%	70.7%
	Tedizolid	45.8%	33.3%	20.8%
	Vancomycin	29.2%	12.5%	58.2%
QUESTION 4				
Which of the following options do you prefer to define the best time to stop combination therapy (shifting to beta-lactam monotherapy) for the treatment of meningitis caused by GAS?	48-72 h	8.3%		
	5-7 days	20.8%		
	I would continue combination therapy until the end of the treatment	41.7%		
	When the patient is clinically and hemodynamically stable	29.2%		

<table>
<tr><td colspan="5">BRAIN ABSCESS</td></tr>
<tr><td colspan="2" rowspan="2">QUESTION 1</td><td colspan="3">LIKERT SCALE</td></tr>
<tr><td>SD/D</td><td>N A/D</td><td>A/SA</td></tr>
<tr><td rowspan="3">How much do you agree on a Likert scale with the indication to use each of the following options as first-line therapy for the treatment of brain abscess caused by GAS?</td><td>Ampicillin</td><td>29.2%</td><td>29.2%</td><td>41.7%</td></tr>
<tr><td>Ceftriaxon</td><td>4.2%</td><td>0%</td><td>95.8%</td></tr>
<tr><td>Vancomycin</td><td>29.2%</td><td>12.5%</td><td>58.2%</td></tr>
<tr><td colspan="2" rowspan="2">QUESTION 2</td><td colspan="3">LIKERT SCALE</td></tr>
<tr><td>SD/D</td><td>N A/D</td><td>A/SA</td></tr>
<tr><td rowspan="2">How much do you agree on a Likert scale with using combined antibiotic therapy (beta-lactam + other agent with activity on GAS) to treat brain abscess caused by GAS in following conditions?</td><td>As first line therapy</td><td>8.3%</td><td>4.2%</td><td>87.4%</td></tr>
<tr><td>Not responding after 48-72 h</td><td>50%</td><td>33.3%</td><td>16.6%</td></tr>
<tr><td colspan="2" rowspan="2">QUESTION 3</td><td colspan="3">LIKERT SCALE</td></tr>
<tr><td>SD/D</td><td>N A/D</td><td>A/SA</td></tr>
<tr><td rowspan="4">How much do you agree on a Likert scale with the indication to use each of the following options as combination therapy with beta-lactams for the treatment of brain abscess caused by GAS?</td><td>Clindamycin</td><td>37.4%</td><td>20.8%</td><td>41.7%</td></tr>
<tr><td>Linezolid</td><td>12.5%</td><td>12.5%</td><td>75%</td></tr>
<tr><td>Tedizolid</td><td>41.7%</td><td>25%</td><td>33.3%</td></tr>
<tr><td>Vancomycin</td><td>20.8%</td><td>12.5%</td><td>66.6%</td></tr>
<tr><td colspan="2">QUESTION 4</td><td colspan="3"></td></tr>
<tr><td rowspan="5">Which of the following options do you prefer to define the best time to stop combination therapy (shifting to beta-lactam monotherapy) for the treatment of brain abscess caused by GAS?</td><td>48-72 h</td><td colspan="3">0%</td></tr>
<tr><td>5-7 days</td><td colspan="3">4.2%</td></tr>
<tr><td>I would continue combination therapy until the end of the treatment (4-6 weeks)</td><td colspan="3">8.3%</td></tr>
<tr><td>When the patient is clinically and hemodynamically stable</td><td colspan="3">8.3%</td></tr>
<tr><td>When the brain MRI shows a significant reduction in the size of the lesion</td><td colspan="3">79.2%</td></tr>
</table>

SEPTIC ARTHRITIS/OSTEOMYELITIS				
QUESTION 1		LIKERT SCALE		
		SD/D	N A/D	A/SA
How much do you agree on a Likert scale with the indication to use each of the following options as first-line therapy for the treatment of septic arthritis/osteomyelitis caused by GAS?	Penicillin	25%	16.6%	58.3%
	Ampicillin	20.8%	8.3%	70.8%
	Ceftriaxon	16.7%	12.5%	70.8%
	Vancomycin	54.2%	8.3%	37.5%
QUESTION 2		LIKERT SCALE		
		SD/D	N A/D	A/SA
How much do you agree on a Likert scale with using combined antibiotic therapy (beta-lactam + other agent with activity on GAS) to treat septic arthritis/ostemyelitis caused by GAS in following conditions?	As first line therapy	45.8%	20.8%	33.3%
	Not responding after 48-72 h	16.6%	12.5%	70.8%
QUESTION 3		LIKERT SCALE		
		SD/D	N A/D	A/SA
How much do you agree on a Likert scale with the indication to use each of the following options as combination therapy with beta-lactams for the treatment of septic arthritis/osteomyelitis caused by GAS?	Clindamycin	4.2%	4.2%	91.7%
	Linezolid	20.8%	16.7%	62.5%
	Tedizolid	45.8%	29.2%	25%
	Vancomycin	41.7%	4.2%	54.2%
QUESTION 4				
Which of the following options do you prefer to define the best time to stop combination therapy (shifting to beta-lactam monotherapy) for the treatment of septic arthritis/osteomyelitis caused by GAS?	48-72 h	4.2%		
	5-7 days	45.8%		
	I would continue combination therapy until the end of the treatment	33.3%		
	When the patient is clinically and hemodynamically stable	16.7%		

IMMUNOGLOBULINS				
QUESTION 1		LIKERT SCALE		
		SD/D	N A/D	A/SA
How much do you agree on a Likert Scale with the indication to use intravenous immunoglobulins as first line therapy for the treatment of iGAS infections?		54.2%	29.2%	16.7%
QUESTION 2		LIKERT SCALE		
		SD/D	N A/D	A/SA
How much do you agree on a Likert Scale with the indication to reserve intravenous immunoglobulins for the treatment of iGAS infections in patients that have the following criteria?	Patients that are hemodynamically unstable	20.8%	29.2%	50%
	Patients that are admitted to intensive care unit	20.8%	20.8%	58.3%
	Patients that have STSS	12.5%	16.7%	70.8%
	Patients that have NF	25%	25%	50%
QUESTION 3				
Which of the following dosages of intravenous immunoglobulins do you prefer in pediatric patients affected by iGAS infections?	1 g/kg on the first day then 0.5 g/kg	58.3%		
	2 g/kg as a single infusion	20.8%		
	Other	20.8%		

STEROIDS				
QUESTION 1		LIKERT SCALE		
		SD/D	N A/D	A/SA
How much do you agree on a Likert Scale with the indication to use steroids for the treatment of otomastiditis caused by GAS in the following situations?	I would never use steroids	33.2%	8.3%	54.1 %
	As first line therapy	70.8%	8.3%	16.6 %
	Only in patients not responding to beta-lactams after 48-72 hours of antibiotic treatment	37.4%	45.8%	12.5 %
QUESTION 2		LIKERT SCALE		
		SD/D	N A/D	A/SA
How much do you agree on a Likert Scale with the indication to use steroids for the treatment of meningitis caused by GAS in the following situations?	I would never use steroids	66.6%	25%	8.2%
	As first line therapy	25%	29.1%	45.8 %
	Only in patients not responding to beta-lactams after 48-72 hours of antibiotic treatment	45.8%	37.5%	16.6 %
	In patients with evidence of edema at the MRI	4.2%	20.8%	75%
QUESTION 3		LIKERT SCALE		
		SD/D	N A/D	A/SA
How much do you agree on a Likert Scale with the indication to use steroids for the treatment of brain abscess caused by GAS in the following situations?	I would never use steroids	66.6%	25%	8.2%
	As first line therapy	66.6%	20.8%	12.5%
	Only in patients not responding to beta-lactams after 48-72 hours of antibiotic treatment	45.8%	37.5%	16.6%
	In patients with evidence of edema at the MRI	4.2%	4.2%	91.6%

ANTIBIOTIC PROPHYLAXIS	
QUESTION 1	LIKERT SCALE

		SD/D	N A/D	A/SA
How much do you agree on a Likert Scale with the following definition of close contacts of pediatric patients affected by iGAS infections?<br>Definition of close contact: This is defined as those who have had prolonged contact with the case in a household-type setting during the 7 days before diagnosis of iGAS infection and up to 24 hours after initiation of appropriate antimicrobial therapy in the index case. Examples of such contacts would be those with an overnight stay in the same household, (including extended household if the case has stayed at another household), pupils in the same dormitory, intimate partners, or university students sharing a kitchen in a hall of residence. For a care home, a close contact is defined as someone sharing a bedroom.		0%	29.2%	70.8%
QUESTION 2		LIKERT SCALE		
		SD/D	N A/D	A/SA
How much do you agree on a Likert Scale with the indication to administrate **antibiotic prophylaxis in the following categories of close contacts** (see definition before) of pediatric patients affected by iGAS infections?	Neonates (< or = 28 days)	12.5%	25%	62.5%
	> or = 65 years	12.5%	16.7%	70.8%
	Immunocompromised	16.7%	8.3%	75%
	Patients with recent surgery	20.8%	41.7%	37.5%
	Household contacts regardless of age	50%	29.2%	20.8%
	Household contacts only if 2 or more confirmed or probable iGAS cases occour in the same family unit	20.8%	25%	54.2%
	Close contacts if 2 or more confirmed or probable iGAS cases occur in a community within 1 month	20.8%	29.2%	50%
	Pregnant women (> or = 37 weeks)	12.5%	33.3%	54.2%
	Post-partum women (< or = 28 days)	12.5%%	29.2%	58.3%
	Individuals with chickenpox active lesions within 7 days prior to diagnosis of iGAS infection in the index case or within 48 hours after commencing antibiotics by the iGAS case, if exposure ongoing	12.5%	16.7%	70.8%
QUESTION 3		LIKERT SCALE		
		SD/D	N A/D	A/SA
How much do you agree on a Likert scale with the indication to administrate **antibiotic prophylaxis to the high risk close contacts** (categories listed in the previous		41.7%	25%	33.3%

question) **only in case of positive pharyngeal swab for GAS**?				
QUESTION 4		LIKERT SCALE		
		SD/D	N A/D	A/SA
How much do you agree on a Likert scale with the following definition of “Appropriate timing to administrate chemoprophylaxis to close contacts” of pediatric patients affected by iGAS infections?<br>Definition: For maximum benefit, chemoprophylaxis should be administered as soon as possible (within 24 hours, and preferably the same day) after eligible contacts are identified and not beyond 10 days after iGAS diagnosis in the index case. Advise GPs to maintain low threshold of suspicion for 30 days in all close contacts.		0%	16.7%	83.3%
QUESTION 5		LIKERT SCALE		
		SD/D	N A/D	A/SA
How much do you agree on a Likert scale with the indication to use each of the following **options as prophylaxis** for high risk close contacts (categories listed in a previous question) of pediatric patients affected by iGAS infections?	Penicillin	33.3%	33.3%	33.3%
	Amoxicillin	4.2%	8.3%	87.5%
	Cephalexin	12.5%	29.2%	58.3%
	Clarithromycin, in case of allergy to beta-lactams	25%	12.5%	62.5%
	Azithromycin, in case of allergy to beta-lactams	25%	16.7%	58.3%
	Clindamycin, in case of allergy to beta-lactams	20.8%	16.7%	62.5%
